# Profiling and Integrated Analysis of Differentially Expressed MicroRNAs as Novel Biomarkers of Hepatocellular Carcinoma

**DOI:** 10.3389/fonc.2021.770918

**Published:** 2022-01-31

**Authors:** Yuwei Xie, Yixiu Wang, Weijie Xue, Hao Zou, Kun Li, Kui Liu, Wei Zhao, Chengzhan Zhu, Jingyu Cao

**Affiliations:** ^1^ Department of Hepatobiliary and Pancreatic Surgery, The Affiliated Hospital of Qingdao University, Qingdao, China; ^2^ Department of Hepatic Surgery, Shanghai Cancer Center, Shanghai Medical College, Fudan University, Shanghai, China; ^3^ Department of Gastrointestinal Surgery, The Affiliated Hospital of Qingdao University, Qingdao, China

**Keywords:** hepatocellular carcinoma, bioinformatics, whole-transcriptome sequencing, microRNAs, biomarkers

## Abstract

Hepatocellular carcinoma (HCC) is a heterogeneous disease that has multiple etiologies. It is the most common primary liver cancer, the sixth highest cause of cancer incidences, and the fourth highest cause of cancer-related deaths. The discovery of new biomarkers for the early detection, treatment, and prognosis of HCC would therefore be extremely useful. This study investigated differentially expressed ribonucleic acid (RNA) profiles by constructing a genome-wide profile of clinical samples. Differential expression analysis identified 1,280 differentially expressed messenger RNAs (dif-mRNAs), 99 differentially expressed microRNAs (dif-miRNAs), 181 differentially expressed long non-coding RNAs (dif-lncRNAs), and 31 differentially expressed circular RNAs (dif-circRNAs). Gene ontology (GO) analysis and Kyoto Encyclopedia of Genes and Genomes (KEGG) path analysis were then conducted on these differentially expressed RNAs, revealing that they were clearly related to cell division, foreign body metabolism, and ribosome assembly. A competing endogenous RNA (ceRNA) network was then constructed based on the regulatory dif-miRNA-dif-mRNA and dif-miRNA-dif-lncRNA relationships. These results were also verified using HCC data from the Cancer Genome Atlas (TCGA); seven dif-miRNAs were verified in clinical samples by real-time quantitative polymerase chain reaction (RT-qPCR). Kaplan-Meier survival analysis revealed that the expression levels of Hsa-miR-1269a, Hsa-miR-421, and Hsa-miR-190b were correlated with overall survival. (*P <*0.05). Survival analysis of clinical samples showed that hsa-mir-1269a, hsa-mir-421 were associated with prognosis (p<0.05).This study revealed the general expression characteristics of specific differentially expressed miRNAs using a ceRNA network constructed from HCC samples. Hsa-mir-1269a, hsa-mir-421 may be promising candidates.

## Introduction

Hepatocellular carcinoma (HCC) is the most common type of primary liver cancer; it is also one of the most common primary malignancies worldwide. According to global cancer statistics, HCC is also the fourth highest cause of cancer death (1–3). HCC usually develops in the context of chronic liver disease; its main causes are alcoholic and non-alcoholic fatty liver, and hepatitis B virus (HBV) and hepatitis C virus (HCV) infections (4). Therefore, it is urgent to better understand HCC tumor biology.

The rapid development of high-throughput next-generation sequencing (NGS) technologies has enabled the complete sequencing of entire genomes, permitting further analysis of the genomic profiles of some cancers (5–7). A recent ribonucleic acid (RNA) sequencing study of HCC found that a new type of vacuolar protein sorting 35 (VPS35) oncogene was significantly reduced in HCC cells. VPS35 plays a carcinogenic role by increasing the sorting and transport of fibroblast growth factor receptor 3 (FGFR3) (8). Recent studies have shown that competitive endogenous RNAs (ceRNAs) can act as natural decoys. The systematic functionalizations of non-coding RNAs (ncRNAs), pseudogenes, and circular RNAs (circRNAs) containing microRNA (miRNA) response elements (MREs) together form a complex miRNA-centered ceRNA network. This network constitutes a common microRNA library that affects gene expression and reveals new mechanisms of RNA interactions (9–12). As a ceRNA mechanism, the long-ncRNA (lncRNA) FAL1 can accelerate cell proliferation and metastasis by competitively binding to miR-1236 (13). However, few studies have used whole-transcriptome sequencing strategies to describe a transcriptional map, which could accurately detect the global gene expression profile.

Here, whole-transcriptome sequencing of tumors and corresponding adjacent non-tumors was conducted for six HCC patients. The expression levels of mRNA, lncRNA, miRNA, and circRNA were then analyzed in the cancer and paracancer groups, and their functional interactions were predicted. In addition, these results were validated using HCC data from the Cancer Genome Atlas (TCGA); a ceRNA network regulatory mechanism was then constructed. This study could offer a new molecular mechanism that could help to reveal the onset and progression of HCC, and its prognosis.

## Materials and Methods

### Patients and Tissue Collection

Cancerous and paracancerous tissue samples were obtained from six male patients with HCC, none of whom had received preoperative radiotherapy or chemotherapy. Patients were selected from the Department of Hepatobiliary and Pancreatic Surgery of the Affiliated Hospital of Qingdao University. All patients participating in the study signed an informed consent form. All specimens were examined for HCC by a pathologist prior to preservation (freezing *via* liquid nitrogen). This research was approved by the Medical Ethics Committee of the Affiliated Hospital of Qingdao University.

### RNA Extraction and Quality Control

Total RNA was extracted from frozen tumor tissue and matched paracancerous tissue using TRIzol reagent (Thermofisher Science; Waltham, Massachusetts, USA). RNA degradation and contamination were monitored using 1.5% Sepharose gel, especially for deoxyribonucleic acid (DNA) contamination. RNA concentration and purity were measured using a NanoDrop 2000 spectrophotometer (ThermoFisher Scientific, Wilmington, DE). RNA integrity was assessed using an Agilent Bioanalyzer 2100 system (Agilent Technologies, CA, USA) with the RNA Nano6000 assay kit.

### Library Construction and Sequencing

RNA samples (2.5 ng) were used as input material to prepare libraries for RNA sequencing. Sequencing libraries were constructed and generated using the NEBNextR UltraTM small RNA Sample Prep Kit for Illumina R (NEB, USA), according to the manufacturer’s instructions. Index codes were added to attribute sequences to each individual sample. Finally, polymerase chain reaction (PCR) products were purified using the AMPure XP system (AMPure XP system); library quality was evaluated using the Agilent Bioanalyzer 2100 system. For clustering and sequencing, index-coded samples were clustered on the cBot cluster generation system, using the TruSeq PE Cluster Kitv3-cBot-HS (Illumina) according to the manufacturer’s instructions. After cluster generation, the library was sequenced on the Illumina platform and reads were generated.

### Screening of Differently-Expressed (Dif)-Messenger RNAs (mRNAs), Dif-miRNAs, Dif-lncRNAs, and Dif-circRNAs

Samples with biological replicates were screened for differences using DEseq software; samples without biological replicates were screened for differences using EBseq software. Dif-mRNA, dif-circRNA, dif-lncRNA, and dif-miRNA were screened using fold changes greater than or equal to one and a false discovery rate (FDR) of <0.05 as screening criteria. Dif-mRNA, dif-lncRNA, and dif-miRNA were analyzed using the R package “clusterProfiler” for Gene Ontology-Biological Process (GO-BP) enrichment analysis and Kyoto Encyclopedia of Genes and Genomes (KEGG) enrichment of differential genes (14–16).

### Protein-Protein Interaction (PPI) Network and Module Analysis of Dif-mRNAs

STRING (version: 11.0; https://www.string-db.org/) was used to analyze the interaction relationships between differential proteins (17). The species was set to human and the import gene set was set to all degrees. To obtain the interaction pairs with the closest interaction relationship, the PPI arguments score was set to 0.99 (high confidence). The PPI network was constructed using Cytoscape software (version 3.8.0; https://cytoscape.org/) and the scores of each node were ranked to obtain the significant nodes. The most important cluster modules in the network were analyzed using Cytoscape’s plugin MCODE (18) (version 2.0.0; PPIMCODE https://apps.cytoscape.org/apps/). The screening threshold was set to 5.0 points, k-core=2, degree cutoff=2, node score cutoff=2, and max depth=100 for screening. GO-BP enrichment analysis was also performed for the important cluster module genes. A GO-BP threshold of *P <*0.05 was considered to imply a significant difference.

### Construction of CeRNA Network

The ceRNA network was constructed using miRDB (V5.0; http://mirdb.org), miRTarBase (V7.0; https://maayanlab.cloud/Harmonizome/resource/MiRTarBase), miRWalk (http://mirwalk.umm.uni-heidelberg.de/) and TargetScan (V7.2; TSCAN.org/VERT_72/). These databases were searched for dif-miRNAs targeting dif-mRNAs to obtain dif-miRNA-dif-mRNA regulatory relationships (19). The dif-miRNA-dif-lncRNA regulatory relationships were obtained by searching for interactions between dif-lncRNAs and dif-miRNAs using the miRcode database (version 11; http://www.mircode.org/), RNAhybrid (https://bibiserv.cebitec.uni-bielefeld.de/rnahybrid/). The dif-miRNA-dif-mRNA and dif-miRNA-dif-lncRNA regulatory relationships were integrated and a composite lncRNA-miRNA-mRNA network was established using Cytoscape, according to the regulatory mechanism of the ceRNA network (20).

### TCGA Liver Hepatocellular Carcinoma (LIHC) Database Validation

The data included 374 tumor specimens and 50 paracancerous normal specimens. The expression matrix and annotation information for mRNA, miRNA, and lncRNA were downloaded and the unpaired t-test in LIMMA software package was used to analyze the differential expression of tumor and normal tissues. A |logFC| value of >1 and a *p* value of <0.05 for mRNA and miRNA were used as cutoff points for the differential expression of lncRNAs. The differential expression results of TCGA data, and the dif-mRNAs, dif-miRNAs, and dif-lncRNAs obtained in this study were subjected to co-expression analysis, and a Venn graph was drawn.

### Real-Time Quantitative PCR (RT-qPCR)

Thirty specimens of HCC tissues or normal liver tissues and histopathologically verified paracancerous tissues were selected from the Department of Hepatobiliary and Pancreatic Surgery, Affiliated Hospital of Qingdao University. Total RNA was extracted from HCC tissues and paraneoplastic tissues using Trizol (Takara). Total RNA was reverse transcribed into complementary DNA (cDNA) using a PrimeScript TM RT kit (Takara). RT-qPCR was performed on a LightCycler 480 using ChamQ Universal SYBR qPCR Master Mix (Vazyme) to determine miRNA expression (21). The study was approved by the ethics committee of Affiliated Hospital of Qingdao University.

### Survival Analysis

Survival analysis of dif-miRNA using the Kaplan-Meier plotter (http://kmplot.com). The results were evaluated according to RT-qPCR. Grouping by 2^-ΔΔCT into high and low expression groups, survival analysis was performed according to hsa-miR-1269a, hsa-miR-421, and hsa-miR-190b.

## Result

### Dif-RNA Analysis

**Figure 1:**
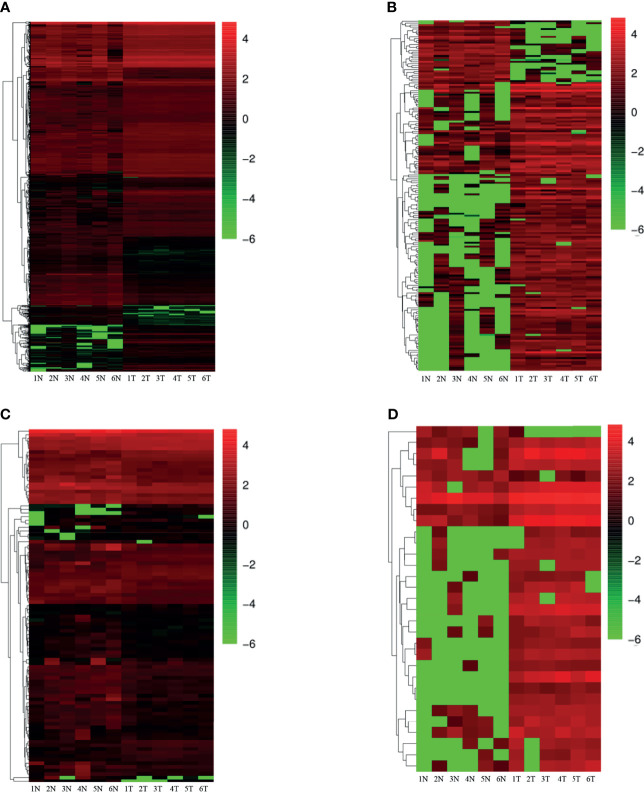
Heatmap of differential expression (N, Normal; T, Tumor) **(A)** differentially expressed mRNAs; **(B)** differentially expressed lncRNAs; **(C)** differentially expressed miRNAs; **(D)** differentially expressed circRNAs. Red indicates up-regulation and green indicates down-regulation.

On the basis of the set screening conditions, a total of 1,280 dif-mRNAs were obtained, of which 904 were up-regulated and 376 were down-regulated. Furthermore, 99 dif-miRNAs were obtained, of which 31 were up-regulated and 68 were down-regulated; 181 dif-lncRNAs were obtained, of which 148 were up-regulated and 33 were down-regulated. Finally, 31 dif-circRNAs were obtained, of which 29 were up-regulated and two were down-regulated. The heat cluster maps of dif-mRNAs, dif-lncRNAs, dif-miRNAs, and dif-circRNAs showed that the tumor samples were clearly divided from the control samples (**Figure 1**), suggesting that the obtained differential expression analysis results were credible.

### Functional Enrichment Analysis of Dif-mRNAs

**Figure 2:**
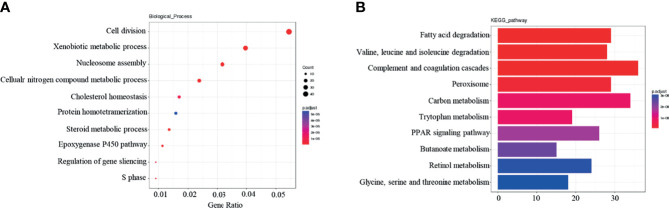
Functional enrichment analysis of Dif-mRNAs: **(A)** Top 10 are enriched for Go-BP terms. **(B)** Top 10 enriched in KEGG pathway.

Following GO and KEGG pathway analysis, the top ten most enriched dif-mRNAs were identified. GO term analysis revealed that the genes displayed were related to biological processes such as cell division, and to the xenobiotic metabolism process (**Figure 2A**). KEGG analysis revealed that these genes were significantly related to fatty acid degradation, complement and coagulation cascades, and peroxisome signaling pathways, among other processes (**Figure 2B**).

### PPI Network and Module Extraction

**Figure 3:**
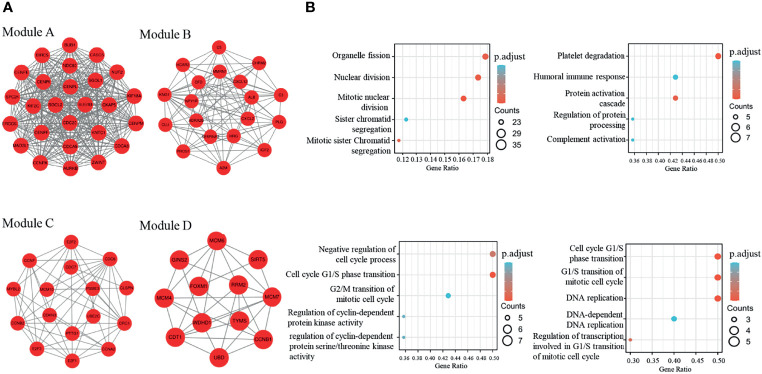
Four modules extracted from the PPI network and GO-BP enrichment analysis of the four modules **(A)** The four modules in the ppi network with a score>5; **(B)** Top5 of GO-BP enrichment analysis of genes in the four modules.

The PPI network of dif-mRNAs consisted of 654 nodes and 2262 mutual pairs, among which nodes with high topological structure scores were considered to be the key nodes. Employing the Cytoscape plug-in MCODE, four sub-network modules (score R ≥5.0) were collected and extracted from the PPI network. Module A (score=26) contained 26 nodes and 325 interaction pairs, module B (score = 10.333) contained 19 nodes and 93 interaction pairs, module C (score = 6.8) contained 16 nodes and 51 interaction pairs, and module D (score=5.273) contained 12 nodes and 29 reciprocity pairs (**Figure 3A**). In addition, Go-BP enrichment analysis of the interacting genes in these modules revealed the top five enrichment results of each module; these were selected for display in order of importance. Among them, the genes of module A were found to be closely related to organelle fission and nuclear division, and the genes of module B were found to be related to platelet degranulation and humoral immune response. The genes in module C were revealed to be related to the negative regulation of the cell cycle process, and to the cell cycle G1/S phase transition. The genes in module D, meanwhile, were found to be related to the G1/S phase transitions of the cell cycle and the mitotic cell cycle (**Figure 3B**).

### Enrichment Analysis of Dif-lncRNA, Dif-miRNA Targeted Regulation Gene

**Figure 4:**
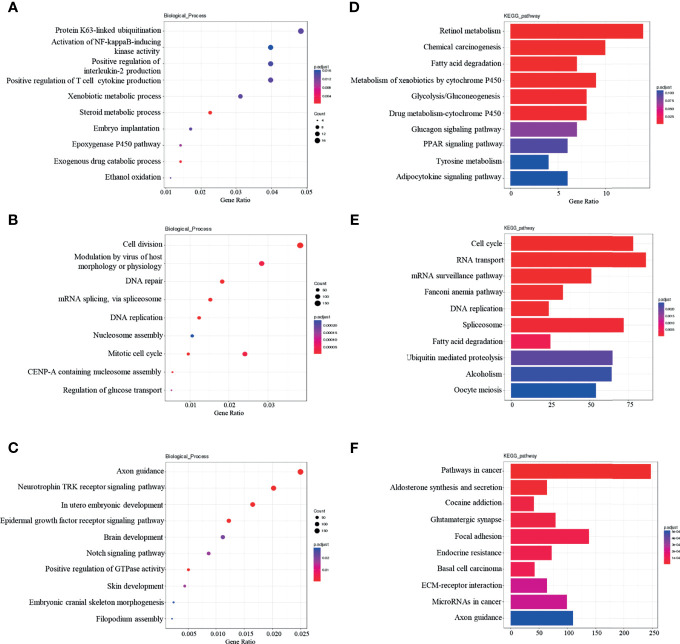
Dif-lncRNAs、Dif-miRNAs GO-BP and KEGG enrichment analysis **(A)** Top10 GO-BP enrichment analysis of cis-lncRNAs target genes; **(B)** Top10 GO-BP enrichment analysis of tran-lncRNAs target genes; **(C)**Top10 GO-BP enrichment analysis of miRNAs target genes; **(D)** Top10 KEGG pathway enrichment analysis of cis-lncRNAs target genes; **(E)** Top10 KEGG pathway enrichment analysis of tran-lncRNAs target genes; **(F)** Top10 KEGG pathway enrichment analysis of miRNAs target genes.

Functional enrichment analysis of dif-lncRNAs (divided into Cis and Trans dif-lncRNAs) target gene and dif-miRNAs target gene was performed, with the top ten richest results being selected. In GO-BP analysis, Cis dif-lncRNA target gene was found to be significantly related to protein k63-linked ubiquitination and the activation of NF-Kappa B-inducing Kinase activity, among other processes. (**Figure 4A**). Tran dif-lncRNA target gene was shown to be associated with processes such as cell division and the viral modulation of host morphology or physiology (**Figure 4B**). Dif-miRNA target gene was revealed to be significantly related to axon guidance and the neurotrophic tyrosine kinase (Trk) receptor signaling pathway, among other processes (**Figure 4C**). KEGG analysis revealed that Cis dif-lncRNA target gene was significantly correlated with retinol metabolism and chemical carcinogenesis (**Figure 4D**) whereas Tran dif-lncRNA target gene was significantly correlated with the cell cycle and RNA transport (**Figure 4E**). Dif-miRNA target gene was revealed to be associated with pathways in cancer, aldosterone synthesis, and secretion (**Figure 4F**).

### CeRNA Network Construction

**Figure 5:**
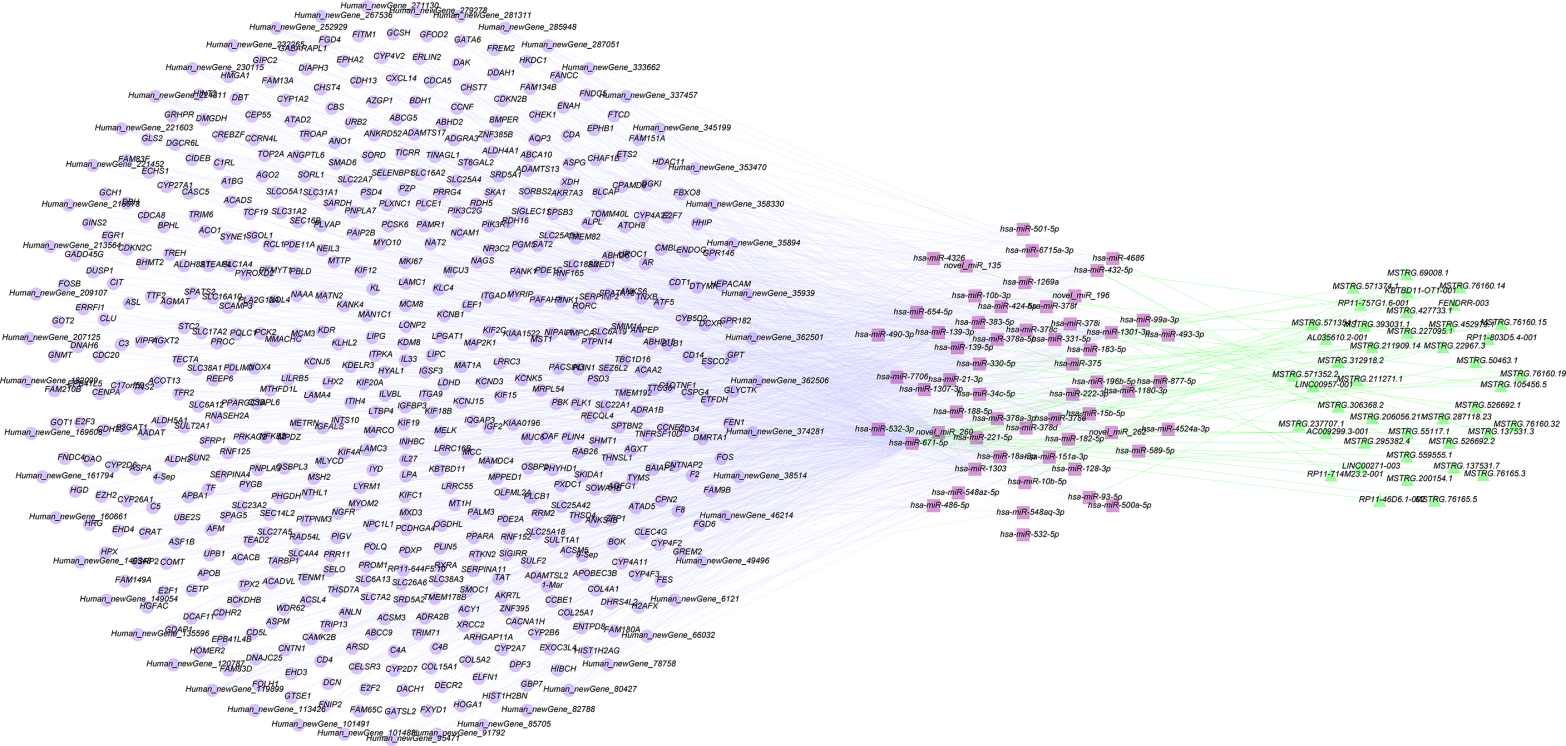
Construction of ceRNA networks by differentially expressed mRNAs, miRNAs and lncRNAs.

According to the mutual regulation formation and information of the dif-miRNA-dif-mRNA and dif-miRNA-dif-lncRNA relationships, lncRNAs and mRNAs that were remarkable differentially expressed and mutually regulated on alike miRNAs were screened. Cytoscape was used to construct the lncRNA-miRNA-mRNA ceRNA network (**Figure 5**).

### TCGA LIHC Data Verification

**Figure 6:**
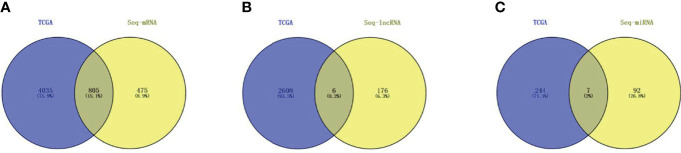
TCGA HCC database and Seq differentially expressed RNAs co-expression analysis **(A)** Dif-mRNAs co-expression analysis; **(B)** Dif-lncRNAs co-expression; **(C)** Dif-miRNAs co-expression.

After setting the screening thresholds at *p <*0.05 and fold change >1, screening the TCGA LIHC dif-mRNA, dif-lncRNA, and dif-miRNA data revealed 4,840 dif-mRNAs, 2,616 dif-lncRNAs, and 251 dif-miRNAs. These were compared with the dif-mRNAs, dif-lncRNAs, and dif-miRNAs obtained from Seq analysis and the dif-lncRNAs and dif-miRNAs identified during co-expression analysis. Thus, 805 dif-mRNAs, 6 dif-lncRNAs, and 7 dif-miRNAs were screened; the resulting vine is shown in **Figures 6A–C**. Among these RNAs, all seven dif-miRNAs (**Table 1**) were up-regulated in HCC tissues.

**Table 1:** Sequence of each miRNA.

hsa-miR-1269a	5´…CUGGACUGAGCCGUGCUACUGG
hsa-miR-421	5´…AUCAACAGACAUUAAUUGGGCGC
hsa-miR-4326	5´…UGUUCCUCUGUCUCCCAGAC
hsa-miR-7706	5´…UGAAGCGCCUGUGCUCUGCCGAGA
hsa-miR-944	5´…AAAUUAUUGUACAUCGGAUGAG
hsa-miR-190b	5´…UGAUAUGUUUGAUAUUGGGUUG
hsa-miR-217	5´…UACUGCAUCAGGAACUGAUUGGA

### RT-qPCR and Survival Analysis

**Figure 7:**
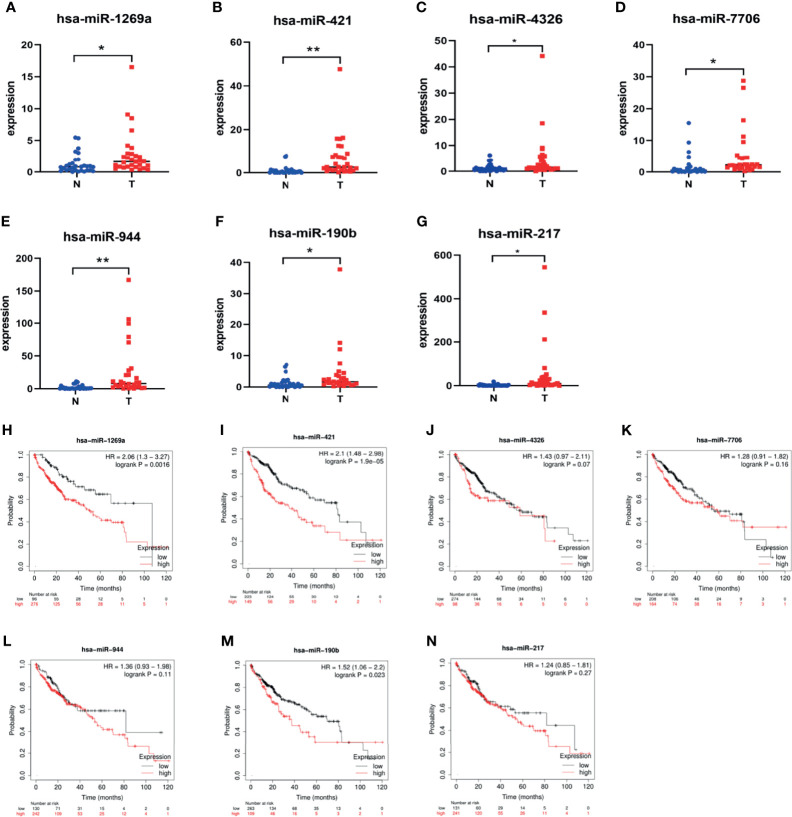
Dif-miRNAs expression in tissue and K-M survival analysis of overall survival (OS) rate **(A)** has-miR-1269a Up-regulation in HCC (*P < 0.05); **(B)** has-miR-421 Up-regulation in HCC (**P < 0.01); **(C)** has-miR-4326 Up-regulation in HCC (*P < 0.05); **(D)** has-miR-7706 Up-regulation in HCC (*P<0.05); **(E)** has-miR-944 Up-regulation in HCC (**P < 0.01); **(F)** has-miR-190b Up-regulation in HCC (*P* < 0.05); **(G)** has-miR-217 Up-regulation in HCC (*P < 0.05); **(H)** Correlation of hsa-miR-1269a with OS (*p* < 0.05); **(I)** Correlation of hsa-miR-421 with OS (*p < 0.05); **(J)** Correlation of hsa-miR-4326 with OS; **(K)** Correlation of hsa-miR-7706 with OS; **(L)** Correlation of hsa-miR-944 with OS; **(M)** Correlation of hsa-miR-190b with OS (*p* < 0.05); **(N)** Correlation of hsa-miR-217 with OS.

**Figure 8:**
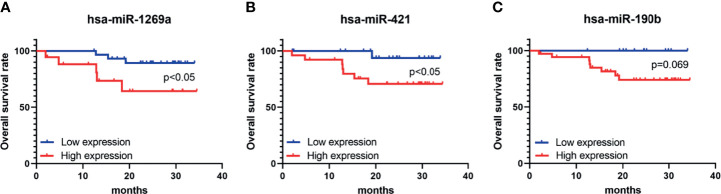
K-M survival analysis of overall survival (OS) of hsa-miR-1269a, hsa-miR-421, and hsa-miR-190b in clinical samples. **(A)** Correlation of hsa-miR-1269a with OS (*p* < 0.05) **(B)** Correlation of hsa-miR-421 with OS (*p* < 0.05) **(C)** Correlation of hsa-miR-4326 with OS.

Seven dif-miRNAs were detected by qRT-PCR, all of which showed significantly higher expression levels in all HCC tissues (**Figures 7A–G**), consistent with the results obtained from the preliminary data. Survival analysis of these seven dif-miRNAs in the Kaplan-Meier database (http://kmplot.com) showed that the overall survival rates of HCC patients with high expression of has-miR-1269a, has-miR-421, and has-miR-190b were significantly lower than those of patients with lower expression of these targets (*p*<0.05 **Figures 7H–N**). Patients were divided into two groups according to RT-qPCR 2^-ΔΔCT: low expression group and high expression group. Subsequent Kaplan-Meier analysis showed that high expression of has-miR-1269a, has-miR-421 was associated with shorter overall survival (*p*<0.05, **Figures 8A–C**).

## Discussion

HCC is one of the most common malignant tumors of the gastrointestinal tract; it is the sixth-most common malignant tumor worldwide, and has the fourth highest mortality rate. Although surgical resection, radiotherapy, chemotherapy, and liver transplantation have all improved significantly in recent years (22, 23), the prognosis remains unsatisfactory. The recurrence rate remains high five years after surgery (24). Therefore, elucidating the molecular mechanisms and processes of HCC is important for discovering new therapeutic targets and improving the clinical prognoses of patients (13).

CeRNA is a usually used to refer to a class of RNAs, incorporating lncRNAs, miRNAs, mRNAs, and circRNAs. These substances can act as sponges after competitively binding to shared miRNAs. CeRNA networks link protein-coding mRNAs to ncRNAs (e.g., miRNAs and lncRNAs) after participating in the development of tumorigenesis (25, 26).

Here, whole transcriptome sequencing of cancer and paraneoplastic tissues from six HCC patients resulted in the identification of 1,280 dif-mRNAs, 99 dif-miRNAs, 181 dif-lncRNAs and 31 dif-circRNAs. Functional enrichment analysis of dif-mRNAs showed that these differential genes were majorly involved in cell division, xenobiotic metabolic processes, and ribosome assembly. A PPI network was constructed, hub genes were analyzed and identified, and GO-BP enrichment analysis was performed for four modules. These four modules were significantly associated with organelle fission, platelet degranulation, the negative regulation of cell cycle processes, and cell cycle G1/S phase transition. GO-BP and KEGG enrichment analyses were performed for dif-miRNAs, dif-lncRNAs target gene. A ceRNA network of lncRNA-miRNA-mRNA was constructed based on the mutual regulatory dif-miRNA-dif-mRNA and dif-miRNA-dif-lncRNA relationships. In addition, most dif-mRNAs and dif-lncRNAs, and a few dif-miRNAs, were successfully verified by TCGA data. The differences highlighted in both sequencing and TCGA data might be related to sample differences or threshold selection. RT-qPCR validation and survival analysis (using the Kaplan-Meier database) was performed for the seven screened dif-miRNAs.

Few studies have used ceRNA networks to assess the prognosis of HCC, and the discovery of potential biomarkers is crucial to improve the diagnoses and prognoses of HCC patients. It has been shown that the ceRNA networks of breast cancer tissues and matched normal tissues differ, with some ceRNAs being activated in cancerous tissues, leading to the development of breast cancer; they are inactive in normal cells, however (27). Those ceRNAs that are differently expressed in cancer and normal tissues could be used as potential markers. It is well known that miRNAs are an integral part of the cancer development process (28).Thus, studies into the prognostic relevance of miRNA regulatory aspects are essential. In this context, here seven co-expressed dif-miRNAs were validated in HCC tissues, consistent with transcriptome sequencing results and differential miRNA expression in TCGA HCC. Then, survival analysis was performed, revealing that Hsa-miR-1269a, Hsa-miR-421, and Hsa-miR-190b were significantly correlated with overall survival (*p <*0.05). It has been found that Hsa-miR-1269a is upregulated in advanced colorectal cancer, and that it forms a regenerative feedback loop with the transforming growth factor-beta (TGF-β) signaling pathway to promote metastasis in colorectal cancer (29). Hsa-miR-1269a has been shown to be highly expressed in uveal melanoma and clear cell renal cell carcinoma, interrelated with poor patient prognosis. Therefore, it can be used as a prognostic effect to predict patient survival (30, 31). In serum exosomes, Hsa-miR-1269a plays an oncogenic role in non-small cell lung cancer (NSCLC); it can be used as a diagnostic marker (32). Hsa-miR-421 has been revealed to be highly expressed in NSCLC. Furthermore, upon binding to kelch-like ECH-associated protein 1 (KEAP1) three prime untranslated region (3’UTR), it has been used to forecast low survival in NSCLC (33). On the contrary, the expression of Hsa-miR-421 expression has been found to be downregulated in breast cancer tissues and metastatic cell lines. Decreased levels of its expression have been observed to be associated with lymph node metastasis, recurrence, metastasis, or TNM staging (34). The low expression of Hsa-miR-190b in breast cancer, meanwhile, has been associated with better prognoses (35). Although dif-miRNAs have been validated to some extent, the interconnections of dif-miRNAs in prediction networks have not been discussed. As previously reported, miRNA-1269a facilitates the proliferation and apoptosis of glioma cells by directly targeting ATRX (36). The lncRNA DLEUI increases the expression of rho associated coiled-coil containing protein kinase 1 (ROCK1) *via* Hsa-miR-421, thereby promoting the progress of papillary thyroid carcinoma (37). Furthermore, the lncRNA tumor suppressor candidate 8 (TUSC8) can inhibit the evolution of papillary thyroid carcinoma *via* the miR-190b myosin regulatory light chain interacting protein (MYLIP) axis, which in turn can inhibit breast cancer growth and metastasis (38). Here, miRNA-1269a was found to target binding to six transmembrane epithelial antigen of prostate 4 (STEAP4) and kinase insert domain receptor (KDR). STEAP4 is associated with adipocyte metabolism and mediates hepatocellular carcinogenesis, whereas KDR is a major growth factor of endothelial cells. It is the main mediator in inducing endothelial cell proliferation, survival, migration and tubule morphogenesis; it is also closely related to tumorigenesis and metastasis.

This study also has some limitations, however. First, it is limited by its small sample size regarding the supply of the general situation of the HCC transcriptome; this may have led to analytical bias. In addition, the functions of the identified dif-miRNAs were not been investigated in depth. The predicted lncRNA-miRNA-mRNA interaction networks need to be further confirmed by performing *in vitro* cellular function experiments, and by using animal models to understand the functions and mechanisms behind them. Researchers are currently investigating the direct functions of identified dif-miRNAs and the molecular mechanisms of targeting RNAs.

## Conclusions

In this study, a ceRNA network was constructed based on whole transcriptome sequencing data. When screened in combination with TCGA, seven differentially expressed miRNAs were identified; further validation revealed that three miRNAs Hsa-miR-1269a, Hsa-miR-421, were significantly associated with prognosis. Therefore, these differential miRNAs are expected to be potential biomarkers or therapeutic targets for the prognosis of HCC.

## Data Availability Statement

The data presented in the study are deposited in the CNGB Sequence Archive (CNSA) of China National GeneBank DataBase (CNGBdb) repository, accession number CNP0002592.

## Ethics Statement

The studies involving human participants were reviewed and approved by Medical Ethics Committee of the Affiliated Hospital of Qingdao University. The patients/participants provided their written informed consent to participate in this study.

## Author Contributions

YX, YW, and WX performed the experiments, designed the research, analyzed the data, and wrote the manuscript. HZ, KunL, KuiL, and WZ analyzed the data, and wrote the manuscript. CZ and JC provided reagents and intellectual guidance. All authors contributed to the article and approved the submitted version.

## Funding

This work was supported by the Taishan Scholars Program of Shandong Province (grant number 2019010668), and the Shandong Higher Education Young Science and Technology Support Program (grant number 2020KJL005).

## Conflict of Interest

The authors declare that the research was conducted in the absence of any commercial or financial relationships that could be construed as a potential conflict of interest.

## Publisher’s Note

All claims expressed in this article are solely those of the authors and do not necessarily represent those of their affiliated organizations, or those of the publisher, the editors and the reviewers. Any product that may be evaluated in this article, or claim that may be made by its manufacturer, is not guaranteed or endorsed by the publisher.
